# New PIN1 inhibitors identified through a pharmacophore-driven, hierarchical consensus docking strategy

**DOI:** 10.1080/14756366.2021.1979970

**Published:** 2021-12-11

**Authors:** Giulio Poli, Miriana Di Stefano, Joan Arias Estevez, Filippo Minutolo, Carlotta Granchi, Antonio Giordano, Salvatore Parisi, Matteo Mauceri, Vincenzo Canzonieri, Marco Macchia, Isabella Caligiuri, Tiziano Tuccinardi, Flavio Rizzolio

**Affiliations:** a Department of Pharmacy, University of Pisa, Pisa, Italy; b Sbarro Institute for Cancer Research and Molecular Medicine, Center for Biotechnology, College of Science and Technology, Temple University, Philadelphia, PA, USA; c Pathology Unit, CRO Aviano, National Cancer Institute, IRCCS, Aviano, Italy; d Department of Molecular Science and Nanosystems, Ca' Foscari University of Venezia, Venezia-Mestre, Italy; e Department of Medical, Surgical and Health Sciences, University of Trieste, Trieste, Italy

**Keywords:** PIN1 inhibitors, virtual screening, molecular modelling, pharmacophore, drug design, cancer

## Abstract

PIN1 is considered as a therapeutic target for a wide variety of tumours. However, most of known inhibitors are devoid of cellular activity despite their good enzyme inhibitory profile. Hence, the lack of effective compounds for the clinic makes the identification of novel PIN1 inhibitors a hot topic in the medicinal chemistry field. In this work, we reported a virtual screening study for the identification of new promising PIN1 inhibitors. A receptor-based procedure was applied to screen different chemical databases of commercial compounds. Based on the whole workflow, two compounds were selected and biologically evaluated. Both ligands, compounds **VS1** and **VS2**, showed a good enzyme inhibitory activity and **VS2** also demonstrated a promising antitumoral activity in ovarian cancer cells. These results confirmed the reliability of our *in silico* protocol and provided a structurally novel ligand as a valuable starting point for the development of new PIN1 inhibitors.

## Introduction

The superfamily of Peptidyl-Prolyl Isomerases (PPIases) catalyses the *cis–trans* isomerisation of peptide bonds *N*-terminal to proline residues in polypeptide chains in order to regulate folding, stability and functions of many proteins. PPIases are subdivided in three families, namely Cyclophilins, FK506 Binding Proteins (FKBPs) and Parvulins, defined based on their structural characteristics. The first two families of PPIases include large enzymes, also called immunophilins, that are inhibited by immunosuppressants like Cyclosporin and FK506^1^. The family of Parvulins, to which PIN1 belongs, comprises smaller PPIases that are not sensitive to these drugs. PIN1 is the only known PPIase that mediates the *cis-trans* conformational switch of phosphorylated Ser/Thr-Pro motifs^2–4^. PIN1 is involved in several cellular processes and pathways, including cell proliferation, division, differentiation, senescence and apoptosis^5^^,^^6^. On the one hand, it acts as a preservative agent of nervous tissue integrity by preventing the onset of neurodegenerative disorders: it regulates amyloid precursor protein processing and amyloid-beta production. Indeed, PIN1 is almost deficient in neurodegenerative diseases, such as Parkinson’s^7^, Alzheimer’s^8^^,^^9^ and Huntington’s disease^10^. On the other hand, PIN1 facilitates multiple cancer-driving pathways^4^^,^^11^. It is highly expressed in most types of cancers, such as breast^12^^,^^13^, prostate^14^, lung, colon and ovarian cancer^15^^,^^16^.

Several PIN1 inhibitors with high potency in biochemical assays are reported in literature, but many of these molecules have limited activity in cells. The development of PIN1 inhibitors began with the discovery of an irreversible small molecule inhibitor, Juglone^17^. Reversible inhibitor design was initially based on the substrate analogue Pintide^18^, which achieved low micromolar potency. Subsequent studies led to the discovery of reversible phosphorylated inhibitors of PIN1, including peptidomimetic and non-peptidomimetic series. The first series of potent, reversible, non-peptidic PIN1 ligands was developed by Pfizer Worldwide Research & Development^19^. Unfortunately, these compounds failed to be active in cell-based assays probably due to the presence of the double negatively charged phosphate group, which mimicked natural phospho-substrates but limited their cell permeability. Liposomal-cyclodextrin encapsulation of one of these inhibitors (LC8) allowed the crossing of this compound through the cell membrane and was effective in killing ovarian cancer cells^16^. A second series of inhibitors was proposed by the Vernalis (R&D) Ltd^20^. These compounds showed IC_50_ values in the submicromolar range, but were still not active in cellular assays. However, a further study led to the synthesis of phenyl-imidazole derivatives endowed with an optimal balance between inhibition activity on PIN1 and antiproliferative activity in prostate cancer cells^21^. Recently, PIN1 was identified as a target of all-*trans* retinoic acid (ATRA). ATRA inhibits PIN1 and leads to its degradation, thereby destabilising PML‐RARα (aberrant promyelocytic leukaemia‐retinoic acid receptor α), to which PIN1 binds, and contrasting the proliferation of acute promyelocytic leukaemia cells. Furthermore, ATRA is able to suppress the growth of triple-negative breast cancer cells via PIN1 inhibition. Despite the efforts made by the medicinal chemistry community, the identification of novel PIN1 inhibitors is still a hot topic in the drug discovery field. In fact, novel studies focussed on the development of PIN1 inhibitors have been recently reported. In particular, a renewed interest for covalent PIN1 ligands emerges from literature, as demonstrated by the recent identification of compound KPT-6566, which impaired growth of lung metastasis *in vivo*^22^, and Sulfopin, which inhibited Myc-driven tumours *in vivo*^23^. Both compounds showed to inhibit PIN1 activity by covalently binding C113 within the enzyme catalytic site. Nevertheless, we recently demonstrated the efficacy of virtual screening (VS) in identifying a novel promising noncovalent, reversible PIN1 inhibitor, compound VS10, which showed antiproliferative activity in four ovarian cancer cell lines and reduced the levels of PIN1 downstream targets, such as β‐catenin, cyclin D1, and pSer473‐Akt^24^.

In this context, a receptor‐based VS study employing pharmacophore modelling, consensus docking and molecular dynamics (MD) simulations was carried out with the aim of discovering structurally novel PIN1 inhibitors. Indeed, the availability of the X‐ray structure of PIN1 in complex with a reference inhibitor allowed the construction of a receptor‐based pharmacophore model used for identifying potential ligands able to establish key interactions with the receptor. Several molecular docking procedures were then employed in a pose and interactions consensus strategy for predicting highly reliable binding dispositions of the filtered ligands within the target protein, which were then refined and further studied through MD simulations. The whole workflow allowed the identification of two novel PIN1 inhibitors to be considered as valuable starting point for hit-to-lead and future lead optimizations.

## Materials and methods

### Pharmacophore model generation and screening

The pharmacophore model was created using Ligandscout 4.2^25^. The pharmacophore hypothesis was built from the X-ray structure of PIN1 in complex with a phenyl-imidazole inhibitor (PDB code: 2XP7)^21^. An exhaustive pharmacophore model including all the possible features identified by the program was constructed and, subsequently, only the desired features were retained in the final pharmacophore model, for a total of 6 features. The selected features included 4 H-bond acceptors, 1 H-bond donor and 1 aromatic feature. Moreover, the excluded volume spheres defined on the basis of the receptor structure, as implemented in the default LigandScout configuration, were added to the model. These represented the region of space that could not be occupied by the ligands during the pharmacophore screening. Approximately 4 million compounds belonging from the Vitas-M, ChemBridge, Enamine and Pharmeks commercial databases were used as the screening database. The software iCon^26^ implemented in LigandScout was used to execute ligand conformational sampling and to set up the screening database. The previously created pharmacophore model, including the excluded volume spheres, was used to filter the generated screening database and to search for compounds with the desired properties. Only the compounds matching the 6 features of the model, which were all set as mandatory, were retrieved and considered for further studies.

### Docking and post-docking analyses

All docking calculations were carried out using the X-ray structure of PIN1 in complex with the phenyl-imidazole inhibitor (PDB code: 2XP7) already employed for pharmacophore modelling. Five different docking procedure were used in this study: Autodock 4.2.3, Glide 5.0 with the standard precision (SP) method and GOLD 5.1 (with ChemScore, GoldScore and ASP fitness functions) employing the procedures previously described^27^^,^^28^. Briefly, for all five docking procedures, the binding cavity used for the docking calculations was defined in order to include all residues which stayed within 10 Å from the centre of the co-crystallized ligand in the reference X-ray complex. Autodock calculations were carried out, performing 20 runs of Lamarckian genetic algorithm for each ligand, with 2500000 steps of energy evaluation, while all other settings were left as their defaults. In all docking studies performed with GOLD, with ChemScore, GoldScore and ASP fitness functions, the “allow early termination” option was deactivated, while the possibility for the ligand to flip ring corners was enabled. The ligands were subjected to 30 genetic algorithm runs and GOLD defaults were used for all other settings. In the docking studies performed with Glide SP, all settings were left as their defaults. The filtering of the docking results was performed by superimposing the docked compounds to the pharmacophore model directly from the supplied poses, without changing their coordinates. The retrieval of compounds matching the six mandatory features of the model was imposed in this search.

### Consensus docking evaluation

The consensus docking evaluation was performed only on the ligands retained after the last docking and post-docking filtering steps, and thus docked into PIN1 binding site using all 5 docking methods. A total of 5 different binding dispositions (top-ranked docking poses) were thus obtained for each compound. The RMSD of each docking pose versus the remaining others was calculated by means of the rms_analysis software of the Gold suite and a 5 × 5 matrix reporting the RMSD results was generated. With the application of an in-house program, the different docking poses relative to each ligand were clustered so that similar docking poses were grouped together. The complete-linkage method was employed as hierarchical clustering algorithm, with an RMSD cut-off of 2.0 Å, thus generating clusters of poses with reciprocal distances in terms of RMSD below 2.0 Å. The consensus level of each ligand was defined as the number of docking methods generating similar binding poses (included within the so obtained clusters).

### MD Simulations

All simulations were performed using AMBER, version 16. General Amber force field (GAFF) parameters were assigned to the ligands, while partial charges were calculated using the AM1-BCC method as implemented in the Antechamber suite of AMBER 16. The complex was placed in a rectangular parallelepiped water box, by using TIP3P explicit solvent model and solvated with a 20.0 Å water cap. Sodium ions were added as counterions to neutralise the system. Before MD simulations, the whole system was energy minimised through 5000 steps of steepest descent followed by conjugate gradient, until a convergence of 0.05 kcal/(mol·Å^2^), imposing a harmonic force constant of 10 kcal/(mol·Å^2^) only on the protein α carbons. The minimised complexes were used as starting conformations for the MD simulations. PME electrostatics and periodic boundary conditions were used in the simulation. The time step of the simulations was 2.0 fs with a cut-off of 10 Å for the non-bonded interactions, and SHAKE was employed to keep all bonds involving hydrogen atoms rigid. A constant volume periodic boundary MD was carried out for 0.5 ns, during which the temperature was raised from 0 to 300 K. The system was then equilibrated through 3 ns of constant pressure simulation, using the Langevin thermostat in order to maintain the temperature of the system constant. Then, additional 6.5 ns of constant pressure MD production were performed. Thus, a total of 10 ns MD simulation was carried out for each protein-ligand complex analysed in this study. All the α carbons of the protein were restrained with a harmonic force constant of 10 kcal/mol·Å^2^ during the whole MD simulation. All the obtained MD trajectories were analysed using the cpptraj program implemented in Amber 16.

### *In vitro* PIN1 inhibition activity

Compounds identified by virtual screening were purchased from Pharmeks (with a purity >92%, as stated by the supplier) and tested for PIN1 inhibitory activity using the *in vitro* fluorescent SensoLyte Green Pin1 Assay Kit (AS-72240; AnaSpec, Fremont, CA, USA). Fluorescent values were recorded with an Infinite M1000 PRO microplate reader (Tecan, Mannedorf, Switzerland). IC_50_ values were determined from logistical dose-response curves and performed in triplicates.

### Cytotoxic assay

Human ovarian OVCAR3, OVCAR5, and SKOV3 cancer cell lines were maintained at 37 °C in a humidified atmosphere containing 5% CO_2_ according to the supplier. Cells (5 × 10^2^) were plated in 96-well culture plates. The day after seeding, vehicle or compounds were added at different concentrations to the medium. Compounds were added to the cell culture at a concentration ranging from 200 to 0.02 µM. Cell viability was measured after 96 h according to the supplier (CellTiter-Glo® luminescence assay, Promega G7571) with a Tecan M1000 PRO instrument. IC_50_ values were calculated from logistical dose-response curves and performed in triplicates.

### Distribution coefficient calculation

The logarithm of the octanol/water distribution coefficient (logD) at pH 7.4, was calculated using the logD plugin of InstantJChem software^29^. The calculation was performed using the consensus method, which combines the ChemAxon's own model derived from Viswanadhan et al.^30^ with and the model from Klopman et al.^31^. All settings were left as their defaults.

## Results and discussion

**Figure 1.**
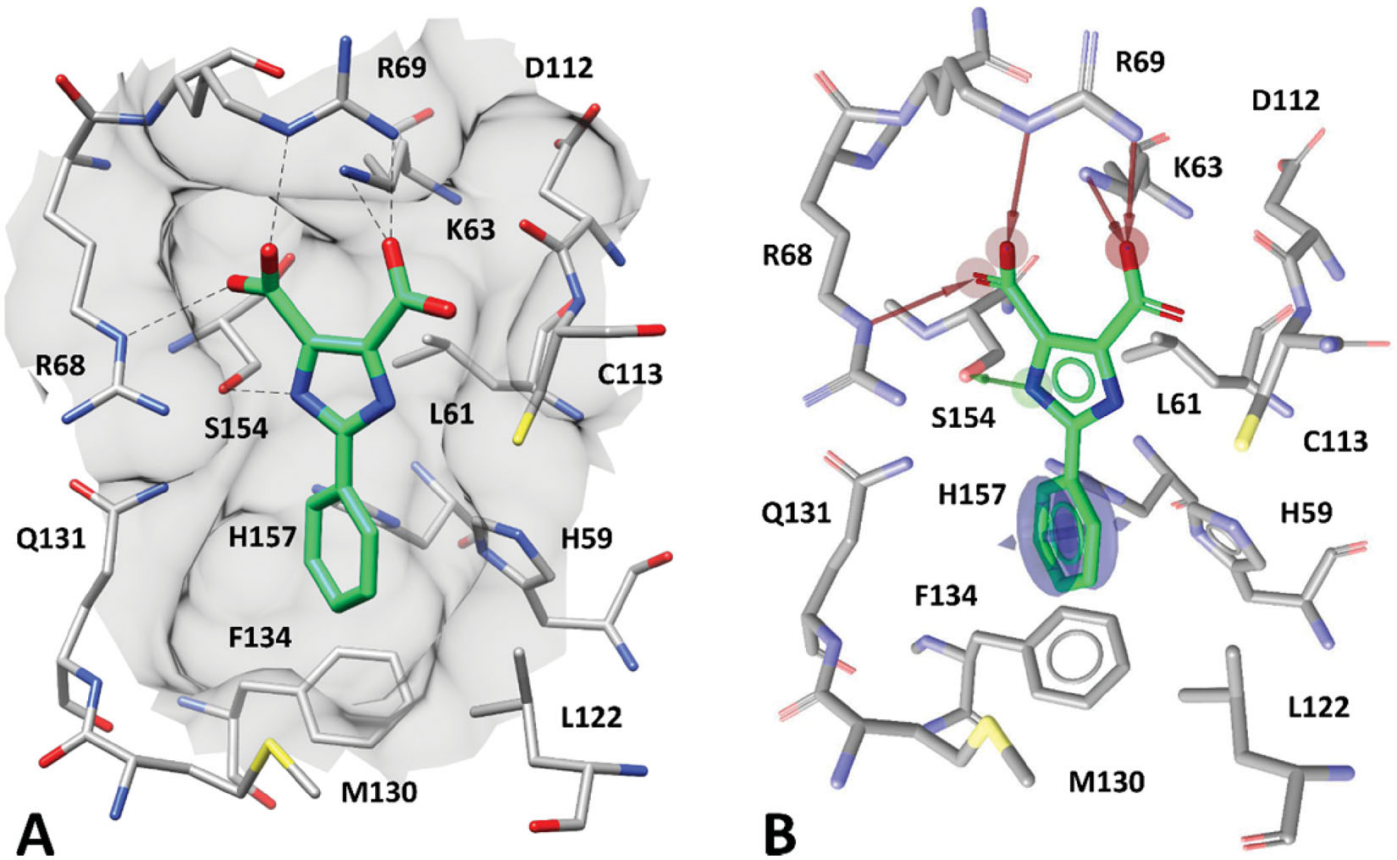
Phenyl-imidazole inhibitor within PIN1 binding site (PDB code: 2XP7). A) The protein residues surrounding the ligand (green), constituting the binding site, are shown as grey sticks, while hydrogen bonds are shown as black dashed lines. The surface of the protein binding site in the surrounding of the ligand is also shown. B) Receptor-based pharmacophore model with the 6 different features superimposed with PIN1 inhibitor and binding site in the X-ray complex.

With the aim of developing a virtual screening (VS) protocol for the identification of new PIN1 inhibitors, the X-ray structure of the PIN1 in complex with a phenyl-imidazole inhibitor (PDB code: 2XP7^21^) was used as a reference for the development of a receptor-based pharmacophore model. The analysis of the crystallographic complex allowed us to identify the main ligand-protein interactions supposed to be responsible for the activity of the inhibitor. The co-crystallized ligand is able to form salt bridges and H-bond interactions with three different residues, i.e. K63, R68 and R69, thanks to its two carboxylic groups (Figure 1(A)). In particular, K63 predominantly interacts with the carboxylic group of the ligand directed towards C113, while the other carboxylic group interacts with R68; finally, both carboxylic groups of the ligand form interactions with R69. Moreover, the compound shows an additional H-bond with S154, established by its imidazole core, and forms multiple aromatic and hydrophobic interactions. In fact, the phenyl ring connected to the imidazole core of the ligand is placed in a shallow, solvent-exposed hydrophobic pocket mainly constituted by H59, L122, M130, Q131, F134 and H157, forming aromatic interactions with H59, F134 and H157, while showing lipophilic interactions with the other residues of the pocket. Based on the main ligand-protein interactions observed in the reference crystallographic complex, a receptor based pharmacophore model was developed using LigandScout software^25^. The generated model comprised a total of 6 pharmacophore features (Figure 1(B)): two H-bond acceptors representing the two interactions with R69, two further H-bond acceptors representing the interactions with K63 and R68, an H-bond donor representing the interaction with S154 and an aromatic feature representing the interactions formed by the phenyl group of the ligand with PIN1 hydrophobic pocket, particularly with H59, F134 and H157.

The receptor-based pharmacophore model was used to screen a database including more than 4 million commercial compounds, with the aim of identifying small-molecules able to form the same key interactions with PIN1 binding site represented by the model. The pharmacophore screening was performed by setting all the features of the model as mandatory, thus identifying compounds that could properly interact with K63, R68, R69, S154 and with the residues forming the hydrophobic pocket of PIN1 binding site. By using this filter only 5576 ligands were selected and subsequently subjected to docking calculations by using five different docking methods, i.e. Autodock 4.2.3, Glide 5.0 with the standard precision (SP) method and GOLD 5.1 (with ChemScore, GoldScore and ASP fitness functions). A hierarchical docking strategy was applied in order to reduce the required computation time. Precisely, the ligands were initially docked using the fastest docking method and then subjected to a post-docking filter, in which only the compounds still matching the six pharmacophore features in their predicted binding mode were further considered; such compounds were thus subjected to the next docking calculations using the second fastest procedure, and so on. This way, progressively smaller numbers of compounds were docked at each step of the hierarchical docking protocol. After the last step, only 35 compounds that complied with the desired pharmacophore features in all five predicted binding dispositions were eventually retained. At this point, the selected compounds were subjected to a pose consensus analysis, as previously performed,^27^ in which an in-house software was used to cluster the five different docking poses obtained for each ligand based on a hierarchical agglomerative clustering method (see Materials and Methods for details). At the end of the clustering process, only compounds for which at least 4 docking poses could be clustered together, showing a reciprocal root-mean-square deviation (RMSD) below 2.0 Å, were further considered. By using this filter, only 10 ligands, for which at least 4 docking methods predicted the same binding orientation, could be selected and further studied through molecular dynamics (MD) simulations aimed at evaluating the stability of their predicted binding mode and key interactions with the protein. The 10 predicted ligand-protein complexes were thus subjected to a 10 ns MD protocol; the results were then analysed in terms of RMSD of ligand disposition during the simulation, with respect to the initial coordinates, as well as in terms of stability of the predicted H-bonds with K63, R68, R69 and S154. Only two compounds, which showed an RMSD of their disposition during the MD below 2.0 Å and maintained at least 3 out of the 5 key H-bonds with PIN1 binding site residues for at least 70% of the MD were considered as novel potential PIN1 inhibitors. These two ligands, **VS1** and **VS2**, were thus selected to be purchased and subjected to biological evaluations. Enzymatic inhibition assays revealed that both compounds were endowed with good PIN1 inhibitory activity, with IC_50_ values of 6.4 and 29.3 µM (Table 1). Notably, the newly identified PIN1 ligands showed a potency in the same range as that of all-*trans* retinoic acid (ATRA, IC_50_ = 33.2 µM), which was used as a reference PIN1 inhibitor for our biological assays.

**Table 1.** Structure and PIN1 inhibition activity of the tested compounds.

#	Structure	IC_50_ (μM)
**VS1**	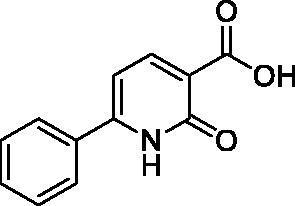	6.4 ± 2.3
**VS2**	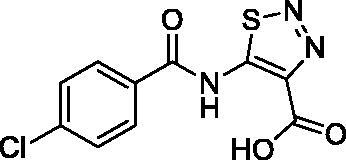	29.3 ± 7.9
**ATRA**	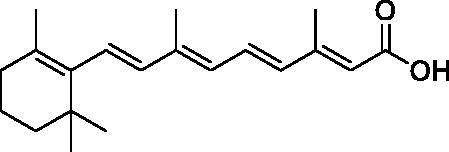	33.2 ± 1.8^a^

^a^See Russo Spena et al. [25].

**Figure 2.**
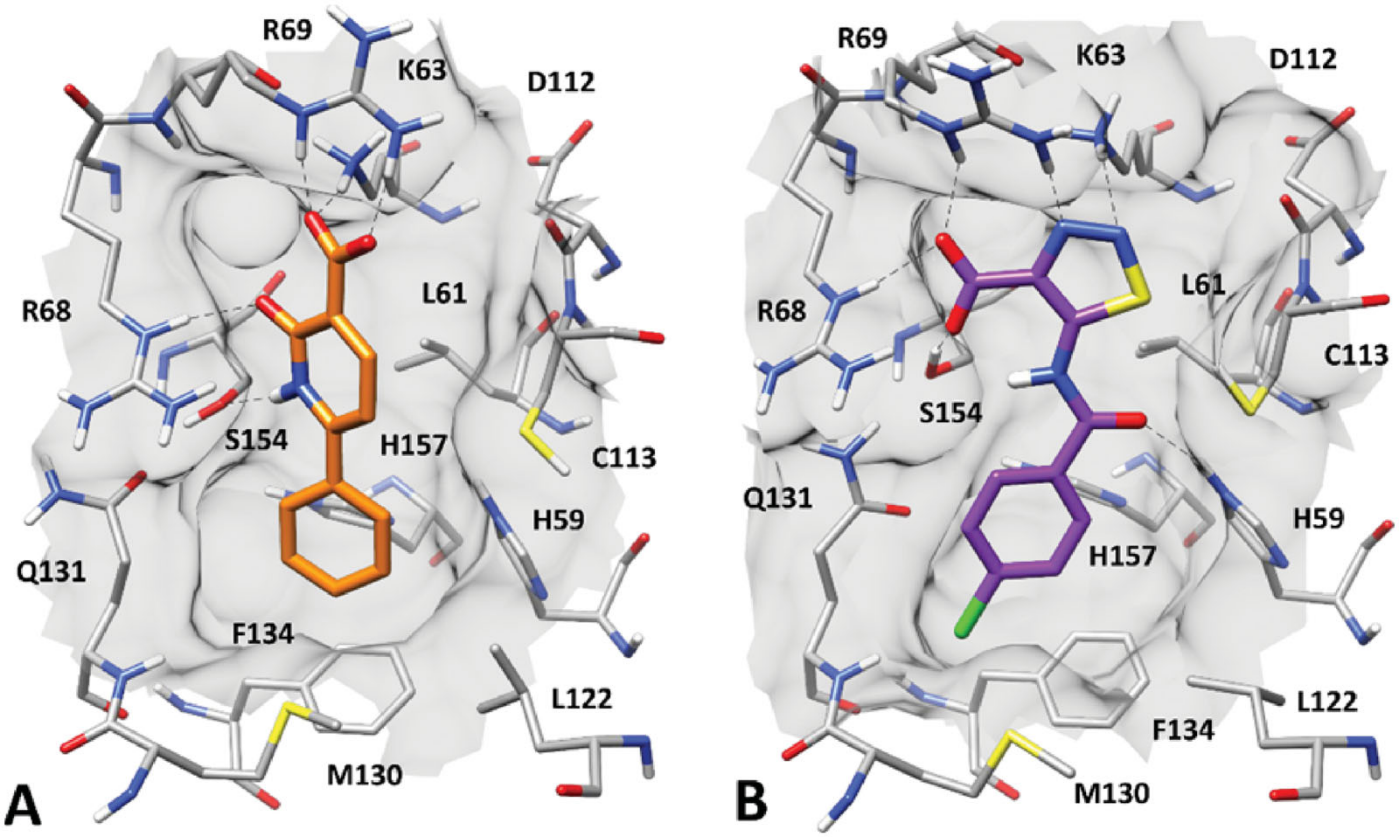
Minimised average structures of (A) compound **VS1** (orange) and (B) compound **VS2** (purple) in complex with PIN1. The protein residues surrounding the ligands, constituting the binding site, are shown as grey sticks, while hydrogen bonds are showed as black dashed lines. The surface of the protein binding site in the surrounding of the ligands is also shown.

Figure 2 shows the binding modes refined through MD simulations predicted for the two VS hits. Compound **VS1** was predicted to form strong salt bridge interactions with both K63 and R69, which were maintained for almost the whole MD, through its carboxylic group (Figure 2(A)). Although the H-bond with R68 appeared to be less stable, the interaction with S154 established by the core pyridone ring of the molecule was found to be maintained for most of the simulation. Moreover, the phenyl group of the ligand, which properly mimics the aromatic ring of the crystallographic phenyl-imidazole PIN1 inhibitor (Figure 1), showed π-π stacking with H59, F134 and H157, as well as lipophilic interactions with L122, M130 and Q131. Finally, the core ring of **VS1** took hydrophobic contacts with L61. Compound **VS2** interacted differently with the charged residues of the binding site, since the main salt bridge interaction formed by the ligand through its carboxylic group, which was maintained for the whole MD simulation, involved R68 (Figure 2(B)). Moreover, this compound interacted with K63 predominantly via the endocyclic nitrogen atoms of its thiadiazole ring, while two different H-bonds with R69 were formed through both the carboxylic and thiadiazole group. Although **VS2** was predicted to form an H-bond with S154 through its amide nitrogen, such interaction was found to be lost during the MD; nevertheless, the carboxylic group of the ligand was able to form a strong H-bond with S154, that was maintained during the whole simulation. Notably, an additional H-bond interaction between the amide C=O group of the ligand and H59, maintained for most of the MD, was also observed. Such interaction may partially compensate for the absence of a salt bridge with K36, as observed for **VS1**, thus justifying the similar activities observed with the two inhibitors. Finally, the *p*-chlorophenyl group of **VS2** was able to form aromatic interactions with H59, F134 and H157, as well as hydrophobic interactions with M130 and Q131.

The two compounds were subjected to further *in vitro* experiments to evaluate their overall antiproliferative activity. Three different ovarian cancer cell lines were treated with increasing concentrations of the tested compounds for 96 h. ATRA was used as the reference compound. As shown in Table 2, only compound **VS2** produced an appreciable inhibition of cell viability in the tested cancer cell lines, with IC_50_ values ranging between 19 and 66 µM. Notably, this compound demonstrated an antiproliferative activity in OVCAR5 cells comparable to that showed by ATRA, while in OVCAR3 and SKOV3 cells **VS2** was found to be about 2-fold and 6-fold more potent, respectively, than the reference compound.

**Table 2.** Antiproliferative activity of compounds **VS1** and **VS2** in various cancer cells.

	IC_50_ (µM, mean ± SD)		
Cell lines	VS1	VS2	ATRA
OVCAR3	> 100	29 ± 4	69 ± 10
OVCAR5	> 100	66 ± 6	69 ± 7
SKOV3	> 100	19 ± 3	112 ± 10

With the aim of rationalising the lack of antiproliferative activity observed for compounds **VS1**, despite its higher PIN1 inhibition potency with respect to **VS2**, the logarithm of the octanol/water distribution coefficient (logD) at pH 7.4, was predicted for both **VS1**, **VS2** and the reference compound ATRA using the logD plugin of InstantJChem software^29^. In fact, the value of logD at physiological pH is known to highly affect the absorption profile of small molecules and, in particular, to be directly correlated with their membrane permeability and *in vitro* apparent permeability^32^^,^^33^. Interestingly, compound **VS2** showed a predicted logD value (-0.32) that was significantly higher than that predicted for **VS1** (corresponding to −2.38) and was closer to the value calculated for ATRA (logD at pH 7.4 = 2.42), which was expected to be the highest among the three compounds due to the enhanced lipophilicity of the retinoid scaffold. This substantial difference in the logD values predicted for the newly identified PIN1 inhibitors may reflect a corresponding difference in the cell membrane permeability profile of the ligands that could justify the absence of antiproliferative activity observed for compound **VS1**, with respect to **VS2**.

In conclusion, the herein reported pharmacophore-driven VS study, based on the combination of receptor-based pharmacophore screening and consensus docking strategies, led to the identification of two novel PIN1 inhibitors with micromolar potency. The results obtained confirmed the reliability of the VS workflow and allowed to expand the chemical space of PIN1 ligands, providing novel compounds structurally different from known inhibitors. In particular, compound **VS2**, which showed a comparable enzyme inhibition and up to 6-fold higher antiproliferative activity in ovarian cancer cells with respect to the reference inhibitor ATRA, could be considered as a valuable starting point for hit-to-lead and future lead optimisation studies aimed at the development of new potent PIN1 inhibitors.
